# ANCA detection with solid phase chemiluminescence assay: diagnostic and severity association in vasculitis

**DOI:** 10.1007/s12026-023-09422-z

**Published:** 2023-09-07

**Authors:** Mónica Renuncio-García, Vanesa Calvo-Río, Fabricio Benavides-Villanueva, Salma Al Fazazi, María Rodríguez-Vidriales, Clara Escagedo-Cagigas, Luis Martín-Penagos, Juan Irure-Ventura, Marcos López-Hoyos, Ricardo Blanco

**Affiliations:** 1 Department of Immunology, Marqués de Valdecilla University Hospital, Avda. Valdecilla S/N, 39008 Santander, Cantabria Spain; 2 Immunopathology Group, Marqués de Valdecilla University Hospital-IDIVAL, 39011 Santander, Spain; 3 Department of Rheumatology, Marqués de Valdecilla University Hospital, 39008 Santander, Cantabria Spain; 4 Department of Rheumatology, Puerta del Mar University Hospital, 11009 Cádiz, Andalucía, Spain; 5 Department of Nephrology, Marqués de Valdecilla University Hospital, 39008 Santander, Cantabria Spain; 6 Molecular Biology Dpt., Universidad Cantabria, Santander, Spain

**Keywords:** ANCA-associated vasculitis, Antibody titer, Severity, Prognosis, Anti-MPO and anti-proteinase 3, Microscopic polyangiitis, Granulomatosis with polyangiitis, Eosinophilic granulomatosis with polyangiitis, Single-organ ANCA vasculitis

## Abstract

ANCA-associated vasculitis (AAV) comprises a group of necrotizing vasculitis that mainly affects small- and medium-sized vessels. Serum anti-neutrophil cytoplasmic antibodies (ANCA), mainly anti-myeloperoxidase (anti-MPO) and anti-proteinase 3 (anti-PR3), levels may correlate to severity, prognosis, and recurrence of the disease. A retrospective analysis of 101 patients with MPO-positive and 54 PR3-positive vasculitis was performed, using laboratory established cut-off value, measured by chemiluminescence. Furthermore, data of renal disease and pulmonary involvement were collected at vasculitis diagnosis, as well as the progress, requiring dialysis, transplant, or mortality. For anti-MPO antibodies with a diagnosis of vasculitis (*n* = 77), an area under the curve (AUC) was calculated (AUC = 0.8084), and a cut-off point of 41.5 IU/ml was determined. There were significant differences in anti-MPO levels between patients with renal or pulmonary dysfunction (*n* = 65) versus those without them (*n* = 36) (*p* = 0.0003), and a cut-off threshold of 60 IU/ml was established. For anti-PR3 antibodies with a diagnosis of vasculitis (*n* = 44), an area under the curve (AUC) was calculated (AUC = 0.7318), and a cut-off point of 20.5 IU/ml was determined. Significant differences in anti-PR3 levels were observed between those patients with renal or pulmonary dysfunction (*n* = 30) and those without them (*n* = 24) (*p* = 0.0048), and a cut-off threshold of 41.5 IU/ml was established. No significant differences between those patients who had a worse disease progression and those who did not were found for anti-MPO and anti-PR3. Anti-MPO and anti-PR3 levels at the moment of vasculitis diagnosis are related with disease severity but not with disease outcome or vasculitis recurrence.

## Introduction

Anti-neutrophil cytoplasmic antibody (ANCA)-associated vasculitis (AAV) is a group of systemic necrotizing vasculitis, which predominantly involve small- and medium-sized vessels (capillaries, venules, arterioles, and small arteries), with few or no immune deposits in affected organs [1].

ANCAs are autoantibodies directed against granular constituents of neutrophils and monocytes, mainly myeloperoxidase (MPO) and proteinase 3 (PR3). However, some patients are diagnosed as ANCA-negative AAV. Those patients could have ANCA that is undetectable with available detection techniques, may have ANCA of as-yet-unidentified specificity, or undergoing pathogenic processes not at all related to ANCA [2].

The main clinical and pathological findings of AAV have been incorporated in the definitions provided by the Chapel Hill Consensus Conference and are used to classify the different syndromes [3].

Those forms of AAV are microscopic polyangiitis (MPA), granulomatosis with polyangiitis (GPA), eosinophilic granulomatosis with polyangiitis (EGPA), and single-organ AAV (for example, renal-limited AAV) [1, 4].

MPA affects systemic small vessels, with commonly necrotizing glomerulonephritis, and the majority of patients with MPA are positive for MPO-ANCAs [5]. Granulomatous inflammation is generally absent. By contrast, GPA is characterized by granulomatous inflammation with necrosis that usually involves the upper and lower respiratory tract and the simultaneous development of necrotizing small- to medium-sized vessel vasculitis. Typically, patients with GPA are positive for PR3-ANCAs. Pauci-immune glomerulonephritis is associated with both MPA and GPA [3, 6, 7].

EGPA is an eosinophil-rich necrotizing granulomatous inflammation that predominantly affects small- to medium-sized vessels. This disease is often involving the respiratory tract such as asthma and allergic sinusitis, and high numbers of eosinophils are present in peripheral blood and affected tissues [8, 9]. Vasculitic manifestations are less frequent and less severe than in GPA and MPA, whereas other features such as cardiomyopathy are more prevalent due to eosinophil-rich organ infiltration. About 50% of patients with EGPA are positive for MPO-ANCAs [2, 7, 10].

Patients with double seropositivity for both MPO and PR3-ANCA are not common and are mainly associated with secondary forms of ANCA-associated vasculitis drugs such as propylthiouracil (an anti-thyroid drug), hydralazine (an anti-hypertension drug), or cocaine. They can induce production of ANCAs and lead to the development of drug-induced AAV [4, 6, 11].

A limited number of studies have evaluated the potential associations between genetic variants and AAV disease severity or disease-related complications. One of them found that for PR3-ANCA vasculitis, carrying a Z allele at SERPINA1 is associated with a significantly higher number of organs involved and higher mortality rates. It has therefore been speculated that deficiency in α1-AT (encoded by the SERPINA1 gene) contributes to a more disseminated and severe disease course. In addition, studying the effect of HLA alleles on disease outcome, for those who had MPO-ANCA-positive vasculitis, HLA-DRB1*0405 was associated with treatment failure. In a recent study, the HLA-DPB1*04:01 allele was associated with risk of relapse among PR3-ANCA-positive patients but not among MPO-ANCA-positive patients [2, 12].

As the expression of ANCA on the cell membrane is triggered by systemic or tissue-specific proinflammatory stimuli (TNF-α, IL-1β, IL-6, IL-18, GM-CSF), involving the complement system, our objective is to determine whether the levels of ANCA induced by immune system stress can be associated with the severity of the disease at diagnosis and used to predict how the disease will progress [6, 11].

## Methods

### Human study and data collection

A retrospective analysis of 101 anti-MPO-positive samples (mean ± standard deviation 64.3 ± 16.7 years) and 54 anti-PR3-positive samples (55.7 ± 13.9 years) obtained at diagnosis was carried out on a hospital in Northern Spain.

Marqués de Valdecilla University Hospital is the unique center that performs the determination of autoantibodies in the Autonomous Community of Cantabria, with a reference population of 580,000 inhabitants.

Demographic and clinical parameters were collected: age of vasculitis diagnostic, sex, symptoms at presentation, affected organ systems, date and first positive ANCA titer, and histological findings. Clinical symptoms include those related to renal disease (hematuria and/or proteinuria) or pulmonary involvement (hemoptysis, asthma, and/or respiratory insufficiency).

In addition, it was evaluated if the disease progressed to the stage of requiring dialysis, kidney or lung transplantation, or if the patient died.

### Study approval

The samples used in this study are part of the collection of samples for research into inflammatory and autoimmune diseases, approved on 27/12/2012 by the Carlos III Health Institute (identification code: C.0001031; principal investigator: Marcos Lopez-Hoyos).

### ANCA determination and statistical analysis

Blood samples were collected from patients with symptoms compatible with vasculitis. Blood was centrifuged for 5 min at 3000 rpm to separate the serum, and anti-MPO and anti-PR3 were determined by chemiluminescence (Bioflash, Werfen). The measuring range for anti-MPO antibodies was 3.2–739.8 CU and for anti-PR3 antibodies was 2.3–3285.3 CU. ANCA were not tested by indirect immunofluorescence (IIF).

Statistical analysis was performed using Graph Pad Prism software 6.0 version. Cut-off points were established following a receiver operating characteristic (ROC) curve. The results were expressed as mean, standard deviation (SD), and percentages. The distribution of continuous variables was assessed using Kolmogorov–Smirnov/Shapiro–Wilk tests. Comparisons were based on non-parametric *U*-Mann–Whitney tests. A two-sided *p*-value < 0.05 was considered statistically significant.

## Results

### Anti-MPO-positive antibodies

Table 1 summarizes the frequency of positive anti-MPO antibodies, using laboratory established cut-off value, in different diseases based on a retrospective investigation of 101 positive anti-MPO antibody patients.

**Table 1:** Diseases associated with positive anti-MPO antibodies (*n* = 101)

Group	Disease	Number (*n*)	Frequency (%)
Vasculitis (*n* = 77)	Microscopic polyangiitis	34	33.7
	*Pauci*-*immune glomerulonephritis*	13	12.9
	Unclassified vasculitis	11	10.9
	Granulomatosis with polyangiitis	10	9.9
	Eosinophilic granulomatosis with polyangiitis	9	8.9
No vasculitis (*n* = 24)	Neoplasm	5	4.9
	Ulcerative colitis	4	3.9
	Infection	3	2.9
	Rheumatoid arthritis	2	1.9
	Intestinal ischemia	2	1.9
	Chron’s disease	1	0.9
	Sjogren’s syndrome	1	0.9
	Autoimmune hepatitis	1	0.9
	Psoriasis	1	0.9
	Lung fibrosis	1	0.9
	Pleural effusion	1	0.9
	Silicosis	1	0.9
	Pachymeningitis	1	0.9

**Fig. 1:**
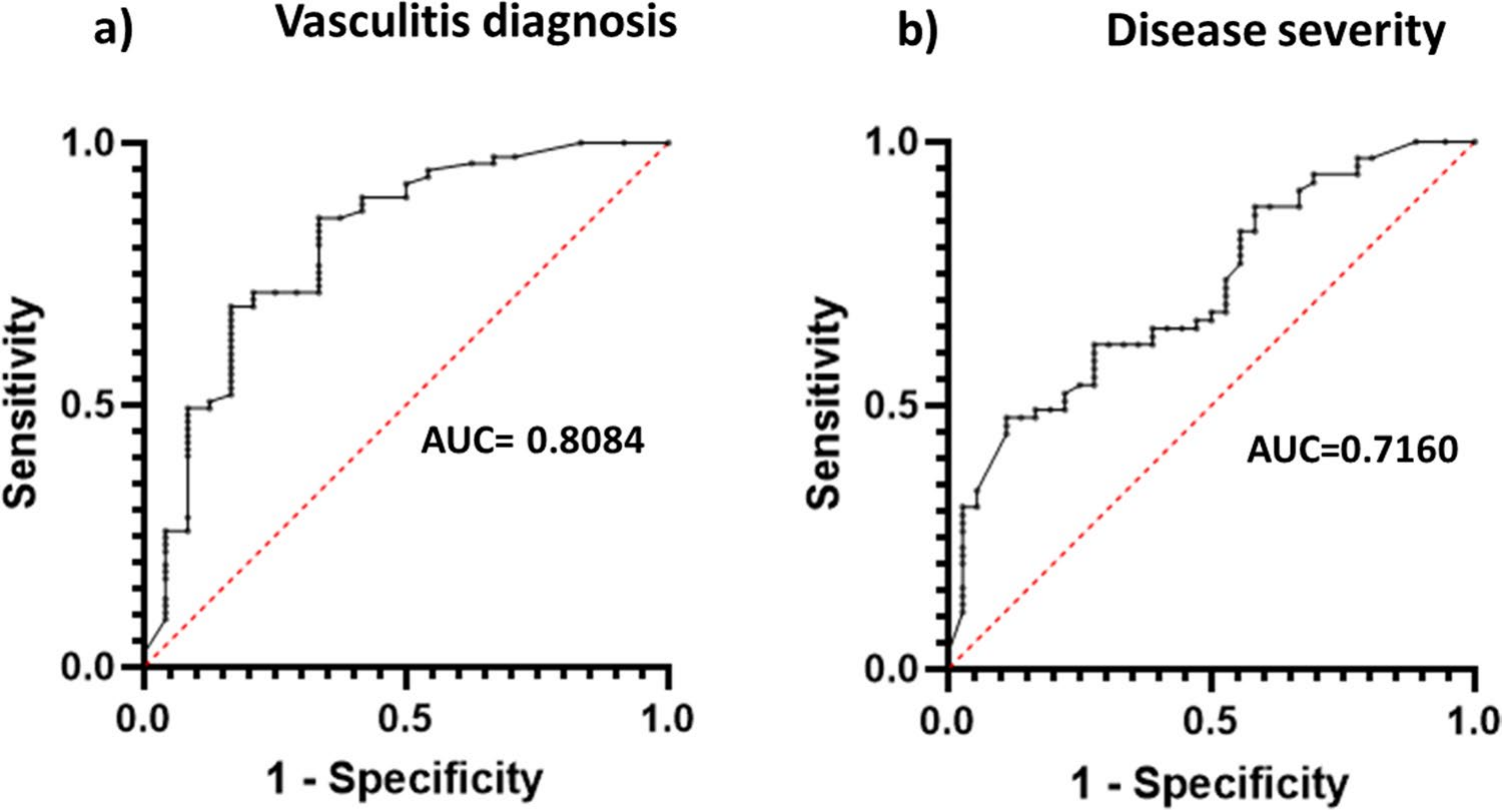
Receiver operating characteristic (ROC) plots for **a** AAV and positive MPO-antibodies (AUC = 0.8084) and **b** AAV and disease severity at diagnosis (AUC = 0.7160)

For anti-MPO antibodies with a diagnosis of vasculitis (*n* = 77), an area under the curve (AUC) was calculated (AUC = 0.8084), and a cut-off point of 41.5 IU/ml was determined (Fig. 1). When the analysis was restricted to exclusively microscopic polyangiitis diagnoses (*n* = 34), the cut-off point was 36.5 IU/ml with an AUC of 0.6435.

**Fig. 2:**
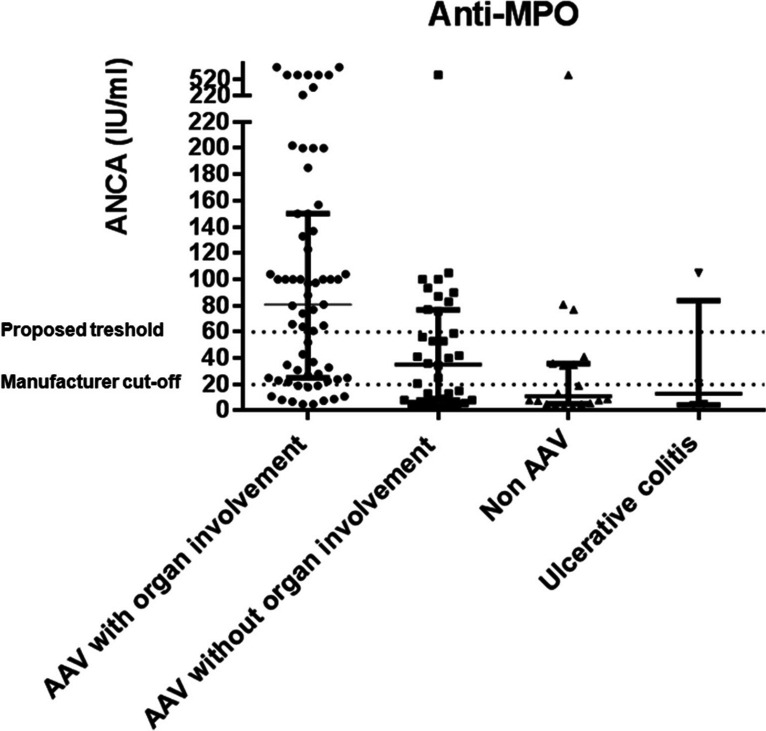
Anti-MPO levels in patients with ANCA associated vasculitis (AAV) and renal/pulmonary involvement (*n* = 65), AAV without renal/pulmonary involvement (*n* = 36), non-AAV except individuals with ulcerative colitis (*n* = 20), and ulcerative colitis patients (*n* = 4). Dot lines represent manufacturer cut-off (20 IU/ml) and the proposed threshold (60 IU/ml)

There were significant differences in anti-MPO levels between patients with renal or pulmonary dysfunction (*n* = 65) versus those without them (*n* = 36) (*p* = 0.0003), and a cut-off threshold of 60 IU/ml was established (Fig. 2).

Finally, after evaluating the illness’s prognosis, an AUC = 0.5546 was found, being no significant differences between those patients who had a worse disease progression (*n* = 19) and those who did not (*n* = 82) (*p* = 0.4643).

### Anti-PR3-positive antibodies

Evaluating the most frequent diseases among patients with positive ANCA, most of the them had GPA, although drug-associated vasculitis was also observed. On the other hand, ulcerative colitis, a digestive pathology associated with anti-PR3 antibodies, was also relatively frequent, as in shown in Table 2.

**Table 2:** Diseases associated with positive anti-PR3 antibodies (*n* = 54)

Group	Disease	Number (*n*)	Frequency (%)
Vasculitis (*n* = 44)	Granulomatosis with polyangeiitis	33	61.1
	Drug-induced vasculitis	5	9.2
	Pauci-immune glomerulonephritis	2	3.7
	Microscopic polyangeiitis	2	3.7
	Large vessel vasculitis	1	1.9
	Unclassified vasculitis	1	1.9
No vasculitis (*n* = 10)	Ulcerative colitis	6	11.1
	Chron’s disease	1	1.9
	Infection	1	1.9
	Diffuse interstitial lung disease	1	1.9
	Hypereosinophil syndrome	1	1.9

**Fig. 3:**
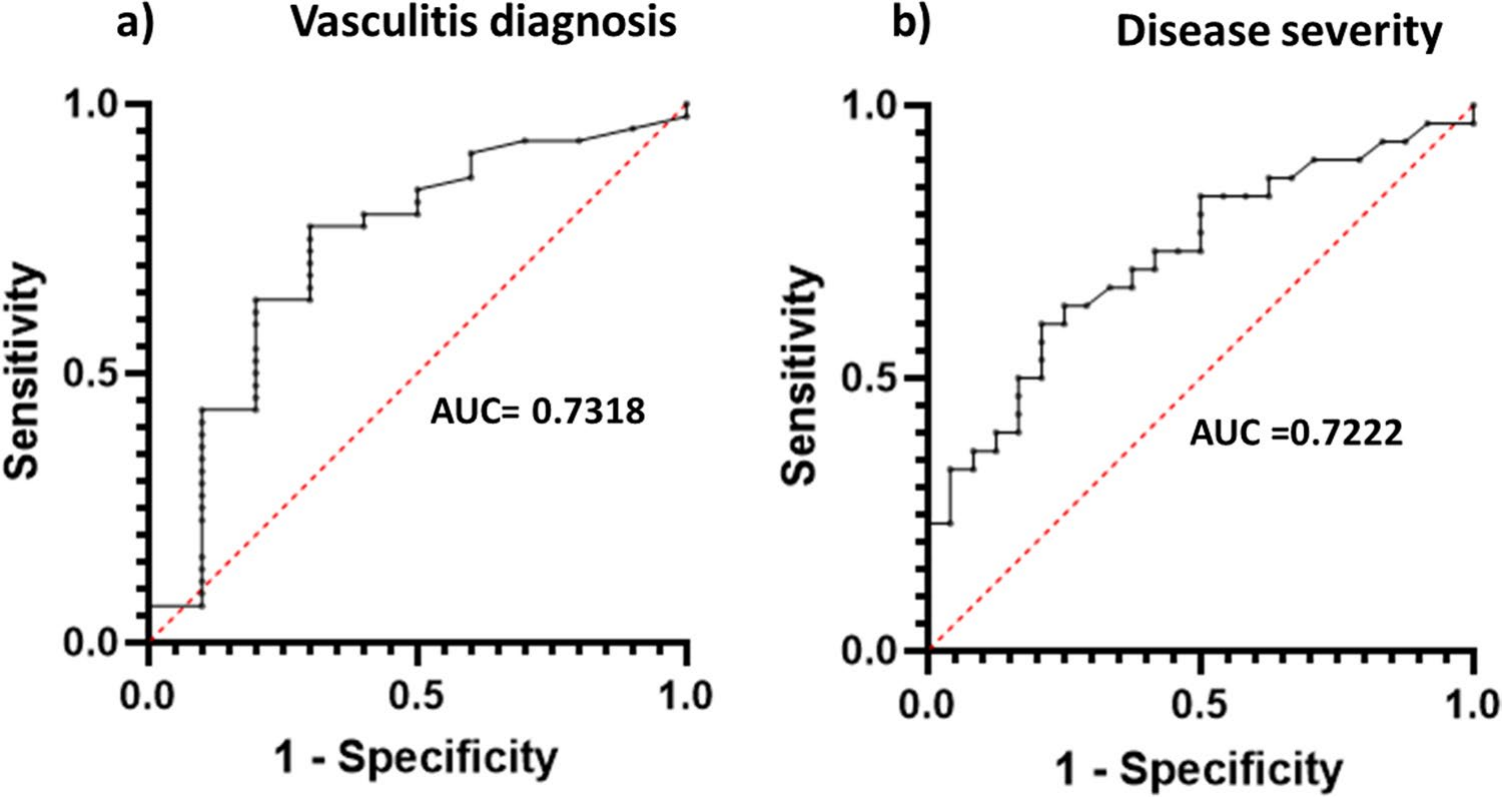
Receiver operating characteristic (ROC) plots for **a** AAV and positive MPO-antibodies (AUC = 0.7318) and **b** AAV and disease severity at diagnosis (AUC = 0.7222)

For anti-PR3 antibodies with a diagnosis of vasculitis (*n* = 44), an area under the curve (AUC) was calculated (AUC = 0.7318), and a cut-off point of 20.5 IU/ml was determined (Fig. 3).

**Fig. 4:**
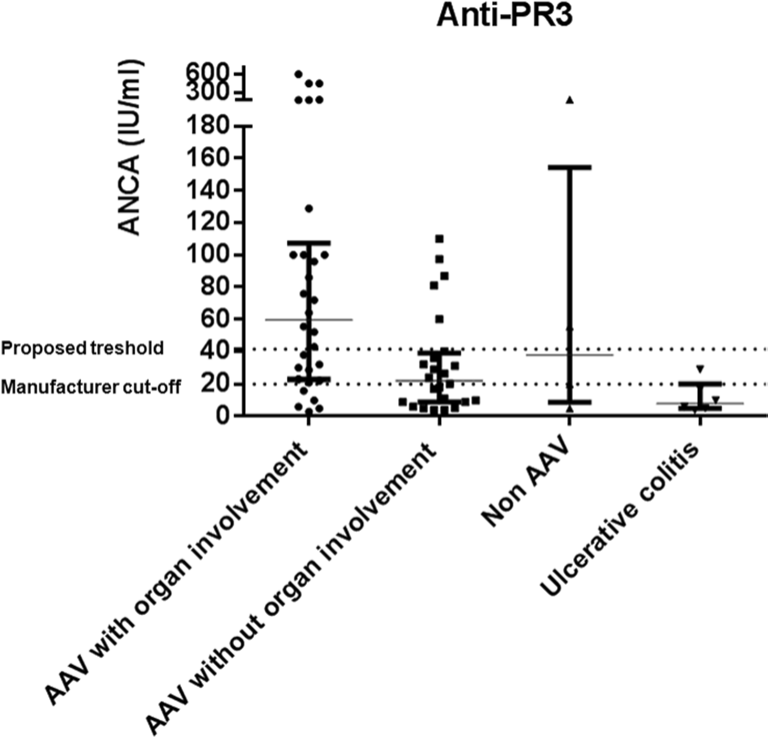
Anti-PR3 levels in patients with ANCA-associated vasculitis AAV and renal/pulmonary involvement (*n* = 30), AAV without renal/pulmonary involvement (*n* = 24), non-AAV except individuals with ulcerative colitis (*n* = 4), and ulcerative colitis patients (*n* = 6). Dot lines represent manufacturer cut-off (20 IU/ml) and the proposed threshold (41.5 IU/ml)

Significant differences in anti-PR3 levels were observed between those patients with renal or pulmonary dysfunction (*n* = 30) and those without them (*n* = 24) (*p* = 0.0048), and a cut-off threshold of 41.5 IU/ml was established (Fig. 4).

Finally, after examining the illness’s prognosis, an AUC = 0.5643 was obtained, being no significant differences between those patients who had a worse disease progression (*n* = 14) and those who did not (*n* = 40) (*p* = 0.4847).

## Discussion

In conclusion, ANCA can be positive in a number of conditions that mimic AAV. There was a strong correlation between the ANCA titers and the clinical diagnosis of AAV in ANCA-positive patients.

Small vessel vasculitis are the most frequent pathologies associated with the presence of ANCA in patient serum. Nevertheless, positive ANCA serology can also be found in other conditions with systemic symptoms, or even in asymptomatic patients [13].

This was consistent with the findings from our study, which reported that 77 of 101 patients with positive anti-MPO antibodies had vasculitis as their primary diagnosis. Thirty-four of these individuals developed MPA, the condition that is most frequently linked to anti-MPO. On the other hand, different illnesses that are not vasculitis are connected to the presence of ANCA in serum. Some of them include chronic infections, autoimmune disorders (such as lupus erythematosus or rheumatoid arthritis), or conditions brought on by medication.

In accordance with the bibliography, in our study, 44 of the 54 patients with anti-PR3 antibodies were diagnosed with vasculitis, being GPA the most prevalent diagnosis. Moreover, vasculitis induced by drugs such as cocaine stands out, sometimes associated with double positive ANCA, but in this case was manifested only with the presence of anti-PR3. Most of the patients who were not diagnosed with vasculitis had ulcerative colitis, an autoimmune digestive disorder associated to ANCA. This finding is in line with the results published by other authors who also showed that PR3-ANCA reactivity is found in a fraction of UC patients when quantified by a sensitive chemiluminescence method, in contrast to other immunoassays, such as FEIA [14].

We found significant differences between patients with renal or pulmonary involvement at diagnosis and the ANCA titer, both for anti-MPO and anti-PR3,* p* = 0.0003 and *p* = 0.0048, respectively.

ANCA titers upon diagnosis were not linked to the prediction of a poor prognosis for the illness, according to our research. The ANCA titer at diagnosis can therefore be used to estimate the disease severity but not the course of the disease.

Some individuals experience continuous disease relapses, so it is crucial to continue looking for indicators that can signal a progression of the condition so that the dose of the treatment can be adjusted properly. In the future, circulating B cell levels and ANCA titers may become the dominant factors guiding maintenance therapy, such as repeated rituximab infusions [15].

### Limitations of the study

Our study has several limitations. First, some vasculitis patients were not completely classified as they did not meet all the criteria. Moreover, not all diagnoses were confirmed by a biopsy due to contradiction or high risk of complications, highly compatible disease, or patient refusal. In addition, some of the diagnoses were recent and a very long follow-up could not be carried out. It would be interesting to increase the number of patients studied and analyze a more detailed follow-up. Finally, another aspect that we consider very relevant and that we have not been able to carry out but that we will take into account for future studies consists of the harmonization of ANCA testing by reporting test result-specific likelihood ratios (LR) in order to improve clinical interpretation of ANCA test results. The use of LR, together with the quantitative result, can be very useful to clinicians, since it provides information on the result regardless of the scale or the cut-off point used for the determination of autoantibodies. If a LR of 10 is reported, this will indicate that the chance to find such value is 10 times higher in patients with AAV than in individuals without AAV [16].

## Data Availability

The data that support the findings of this study are available on request from the corresponding author.

## References

[1] Kronbichler A, Lee KH, Denicolò S, Choi D, Lee H, Ahn D (2020). Immunopathogenesis of ANCA-associated vasculitis. Int J Mol Sci.

[2] Trivioli G, Marquez A, Martorana D, Tesi M, Kronbichler A, Lyons PA (2022). Genetics of ANCA-associated vasculitis: role in pathogenesis, classification and management. Nat Rev Rheumatol.

[3] Jennette JC, Falk RJ, Bacon PA, Basu N, Cid MC, Ferrario F (2013). 2012 revised International Chapel Hill Consensus Conference nomenclature of vasculitides. Arthritis Rheum.

[4] Jain K, Jawa P, Derebail VK, Falk RJ (2021). Treatment updates in antineutrophil cytoplasmic autoantibodies (ANCA) vasculitis. Kidney360..

[5] Sebastiani M, Manfredi A, Vacchi C, Cassone G, Faverio P, Cavazza A (2020). Epidemiology and management of interstitial lung disease in ANCA-associated vasculitis. Clin Exp Rheumatol.

[6] Nakazawa D, Masuda S, Tomaru U, Ishizu A (2019). Pathogenesis and therapeutic interventions for ANCA-associated vasculitis. Nat Rev Rheumatol.

[7] Yates M, Watts R (2017). ANCA-associated vasculitis. Clin Med (Lond).

[8] Sacoto G, Boukhlal S, Specks U, Flores-Suárez LF, Cornec D. Lung involvement in ANCA-associated vasculitis. Presse Med. 2020; 49:104039. 10.1016/j.lpm.2020.104039 32650042

[9] Tedesco M, Gallieni M, Pellegata F, Cozzolino M, Alberici F (2019). Update on ANCA-associated vasculitis: from biomarkers to therapy. J Nephrol.

[10] Furuta S, Iwamoto T, Nakajima H (2019). Update on eosinophilic granulomatosis with polyangiitis. Allergol Int.

[11] Lötscher F, Krusche M, Ruffer N, Kubacki T, Person F, Kötter I (2019). Cocaine-induced ANCA-associated renal disease: a case-based review. Rheumatol Int.

[12] Ross C, Makhzoum JP, Pagnoux C (2022). Updates in ANCA-associated vasculitis. Eur J Rheumatol.

[13] Houben E, Bax WA, Van Dam B, Slieker WAT, Verhave G, Frerichs FCP, et al. Diagnosing ANCA-associated vasculitis in ANCA positive patients: a retrospective analysis on the role of clinical symptoms and the ANCA titre. Medicine. 2016;95:e5096. 10.1097/MD.0000000000005096 PMC5059091 27749588

[14] Mahler M, Damoiseaux J, Ballet V, Dillaerts D, Bentow C, Tervaert JWC (2017). PR3-anti-neutrophil cytoplasmic antibodies (ANCA) in ulcerative colitis. Clin Chem Lab Med.

[15] Koh JH, Kemna MJ, Cohen Tervaert JW, Kim WU (2016). Editorial: Can an increase in antineutrophil cytoplasmic autoantibody titer predict relapses in antineutrophil cytoplasmic antibody-associated vasculitis?. Arthritis Rheumatol.

[16] Bossuyt X, Damoiseaux J, Rasmussen N, Van Paassen P, Hellmich B, Baslund B (2020). Harmonization of antineutrophil cytoplasmic antibodies (ANCA) testing by reporting test result-specific likelihood ratios: position paper. Clin Chem Lab Med.

